# Expansive Open-Door Cervical Laminoplasty: In Situ Reconstruction of Extensor Muscle Insertion on the C2 Spinous Process Combined With Titanium Miniplates Internal Fixation

**DOI:** 10.1097/MD.0000000000001171

**Published:** 2015-07-17

**Authors:** Zhaohui Cheng, Weishan Chen, Shigui Yan, Wanli Li, Shengjun Qian

**Affiliations:** From the Department of Orthopedic Surgery, The Second Affiliated Hospital, School of Medicine, Zhejiang University, People's Republic of China.

## Abstract

Retrospective cohort study.

To evaluate efficacy and relevant problems of in situ reconstruction of extensor muscle insertion on the C2 spinous process combined with titanium miniplates internal fixation in expansive open-door cervical laminoplasty in order to improve surgical treatment effect.

Expansive open-door cervical laminoplasty has been widely applied in clinical practice, but there are a series of postoperative problems. Therefore, decreasing postoperative complications in order to more effectively relieve symptoms remains a subject for additional research.

From October 2011 to September 2013, a total of 60 patients who suffered cervical canal stenosis were treated by expansive open-door laminoplasty with in situ reconstruction of extensor muscle insertion on the C2 spinous process combined with titanium miniplates internal fixation. Changes of cervical curvature index (CI) and range of motion (ROM) were calculated using data from preoperative and postoperative cervical spine X-ray examinations. Clinical function was scored using the Japanese Orthopedics Association Scoring System (JOA) and the neck disability index (NDI).

The mean CI before the operation and at 1-year postoperation were 10.49% ± 3.93% and 14.14 ± 2.85 (*P* < 0.05). The mean ROM values were 43.35 ± 7.55 before the operation, 34.83 ± 7.41 at 1-year postoperation (*P* < 0.05). The NDI scores decreased from 19.42 ± 4.12 to 7.37 ± 2.58, and the JOA scores increased from 8.87 ± 1.99 to 13.55 ± 1.72, representing significant improvement (*P* < 0.05). One patient had postoperative C5 nerve root palsy and completely recovered 1 month later. Neither collapse nor door closure in the open-door side occurred in any of the patients.

Expansive open-door cervical laminoplasty with in situ reconstruction of extensor muscle insertion on the C2 spinous process combined with titanium miniplates internal fixation is a safe and effective surgical method, and can effectively decrease postoperative complications and achieve satisfactory clinical results.

## INTRODUCTION

Expansive open-door cervical laminoplasty is a very important surgical method for the treatment of cervical spondylosis. It is currently used primarily in the treatment of cervical spinal stenosis, including cervical spondylotic myelopathy and ossification of posterior longitudinal ligament. Because its operation is relatively simple and can obviously relieve clinical symptoms, it has been widely used in clinical practice.^1–3^ Ideal expansive laminoplasty cannot only achieve sufficient spinal canal expansion, but also can make the postoperative biomechanical result the same as that of a normal cervical spine without losing range of motion (ROM) and stability. However, current surgical methods have a series of postoperative problems, such as axial symptoms, C5 nerve root palsy, cervical spinal segmental instability, cervical spinal ROM decrease, postoperative door reclosure on door-openness side, and more.^4,5^ Therefore, regarding maintaining cervical spinal stability and ROM, reduction of postoperative complications and more effective relief of symptoms are still a matter of concern. From October 2011 to September 2013, the authors treated 60 cervical canal stenosis patients using expansive open-door cervical laminoplasty with in situ reconstruction of extensor muscle insertion on the C2 spinous process combined with titanium miniplates internal fixation. The patients were followed for 18 to 24 months and all achieved satisfactory clinical efficacy.

## OBJECTIVES AND METHODS

### General Information

From October 2011 to September 2013, a total of 60 patients who suffered cervical canal stenosis were treated by expansive open-door cervical laminoplasty with in situ reconstruction of extensor muscle insertion on the C2 spinous process combined with titanium miniplates internal fixation. These patients included 39 cases of cervical spondylotic myelopathy and 21 cases of ossification of posterior longitudinal ligament. Forty three cases were males and 17 were females, with ages ranging from 36 to 72 years, with an average age of 54.5 years. Written informed consent was obtained from all patients, and the study protocol was approved by the Ethics Committee of The Second Affiliated Hospital, School of Medicine, Zhejiang University. Postoperative follow-up lasted for 18 to 24 months with an average of 20 months. Imaging examinations in all patients included preoperative X-ray, computerized tomography, and magnetic resonance imaging examinations. Follow-up was performed 1 week after operation and 1 year after operation. At each follow-up a cervical spine X-ray examination was performed, and 1 year after the operation a conventional cervical spine MRI examination was performed.

### Surgical Method and Postoperative Treatment

**FIGURE 1:**
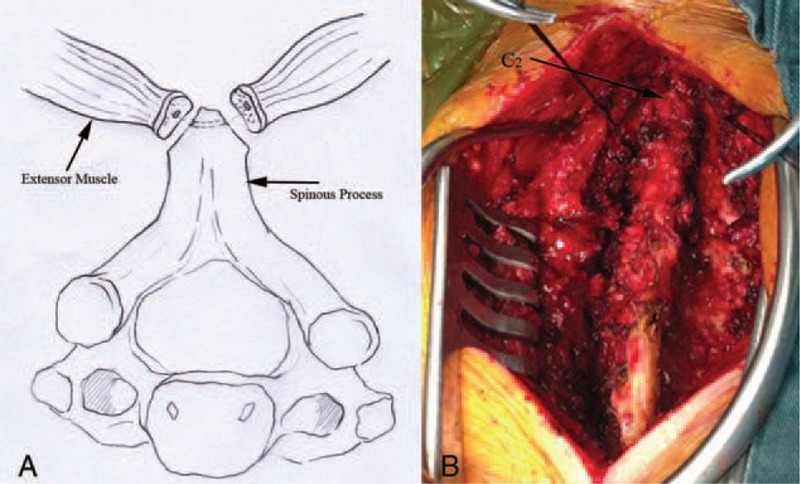
(A) Anatomical diagrammatic figure: bilateral cervical extensor attachment points were split from C2 spinous process (B) Surgical procedure figure.

**FIGURE 2:**
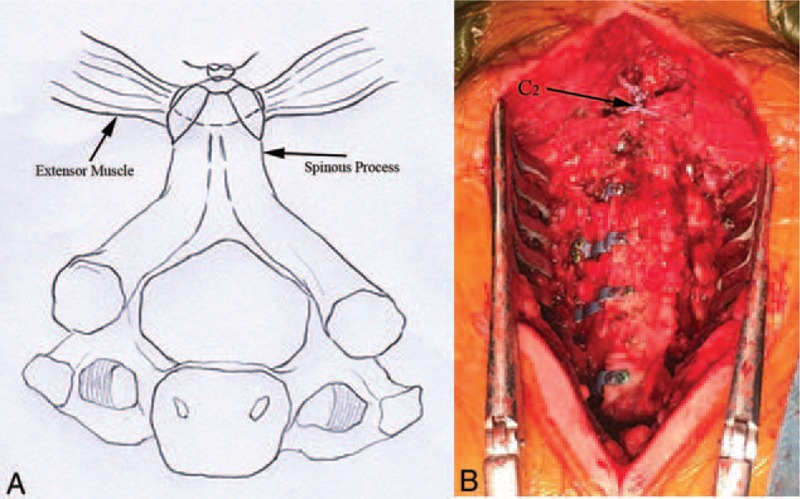
(A) Anatomical diagrammatic figure: bilateral cervical extensor attachment points were refixed on C2 spinous process, (B) Surgical procedure figure.

General anesthesia with tracheal intubation was performed. The head was supported by a Mayfield head holder and the neck was maintained in slight flexion position. C2–T1 spinous process median incision was performed. Along C3–C7 spinous processes and laminas, bilateral paraspinal muscles were subperiosteally freed to the lateral edge of the lateral mass. Interspinous ligament was cut off in C2–C3 and C3–T1, and a pair of spinous process scissors was used to transversely cut off C3–C7 spinous processes from their roots. A spinous process punch was used to drill the middle part of C2 spinous process, and a 1 mm cylindrical burr was used to completely cut off the bilateral cervical extensor attachment point in C2 spinous process along with partial bone fragments (Figure 1 for diagrammatic surgical procedures). The left side was the open-door side and the right side was the hinge side, with the slotting site located on the medial margin of the facet joints. On the hinge side a 2 mm burr was used to grind the whole thickness of the lateral lamina cortex; on the open-door side a 3 mm burr was used to grind the whole thickness of medial and lateral lamina cortices. A 1 mm laminectomy rongeur was used to carefully separate adhesions between ligamentum flavum and dura mate spinalis. C3–C7 laminas were overturned to the right side and were opened; recovery of dura mate spinalis pulse was observed. After the whole spinal canal was opened, titanium miniplates were placed on the open-door side (provided by Shanghai Sanyou Medical Device Company, Jiading District, Shanghai, China), and screws were fixed onto lateral masses and open-door laminas, respectively. The split bilateral cervical extensor attachment points were refixed on C2 spinous process with the suture replacing wire (Figure 2 for diagrammatic surgical procedures). Drainage tubes were routinely placed in all operations and were removed 48 hours after operations. Each of the patients wore a soft collar on the neck for 2 weeks after the operation in order to limit cervical spinal flexion and extension. In addition, the patients were encouraged to begin performing rehabilitation exercise of neck muscles immediately after removal of drainage tubes.

### Observation Indicators and Evaluation Methods

#### Imaging Evaluation

**FIGURE 3:**
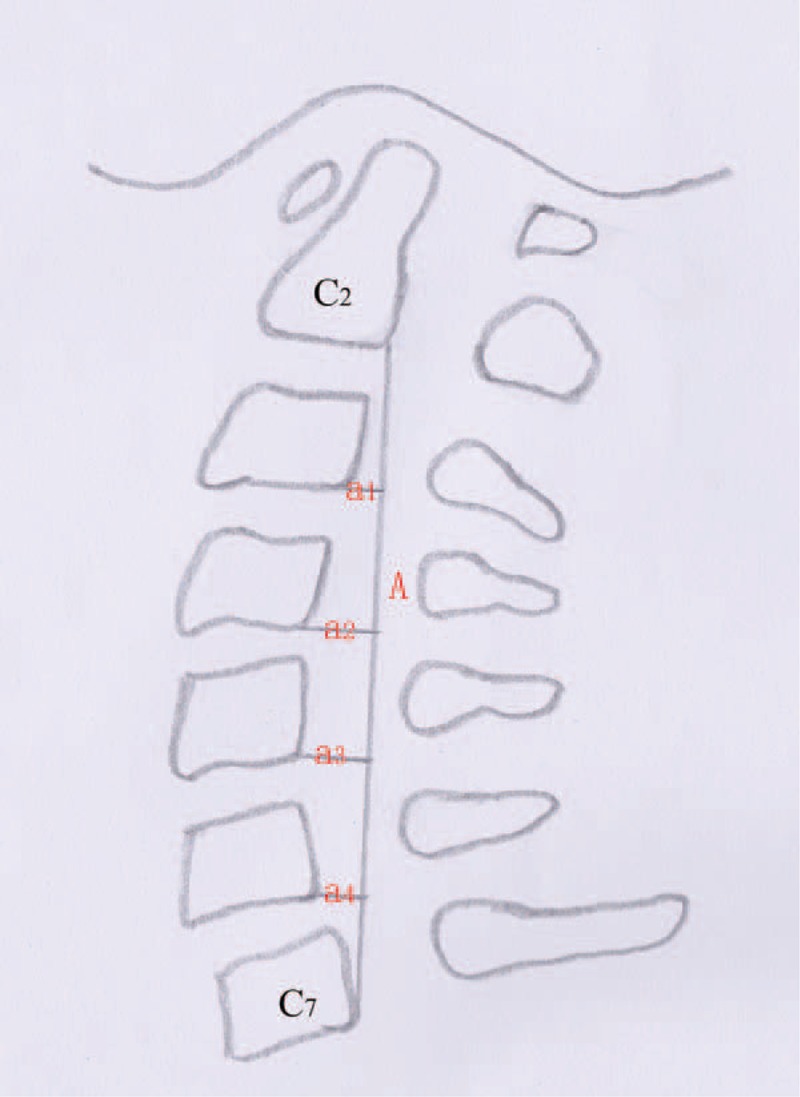
Line segment A is drawn between the postero-inferior edge of the C2 and C7 spinal bodies. The 4 lines, a1–a4, are drawn perpendicular to line A from each postero-inferior edge of the C3, C4, C5, and C6 vertebral bodies. The cervical curvature index (Ishihara) is the percentage of the sum of the 4 segments divided by line segment A that is, =100 × (a1 + a2 + a3 + a4)/A.

**FIGURE 4:**
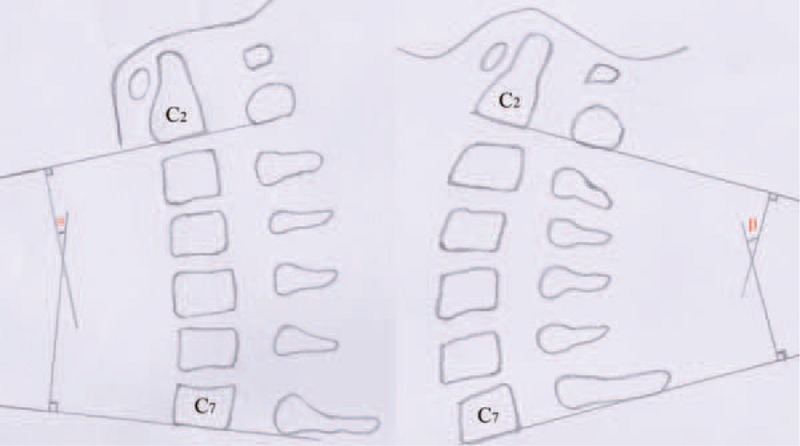
The cervical spine angle (C2–C7) is made by the 2 perpendicular lines of the inferior endplate of C2 and C7.

Cervical curvature and ROM changes were evaluated according to preoperative and postoperative X-ray examinations. The Ishihara method^6^ was used to calculate cervical curvature index (CI) reflecting cervical curvature change (Figure 3). Smaller CI values indicate smaller cervical curvature, and even show if cervical kyphosis has occurred. ROM change in the cervical spine was measured by the Cobb angle.^7^ This angle in flexion position was α and the angle in hyperextension position was β; if cervical spine had reverse flexion (kyphosis), α was negative, that is (ROM = α + β) (Figure 4). One year after the operation, MRI examinations were used to observe the spinal cord compression status on the open-door side.

#### Clinical Function Evaluation

Clinical function evaluation was performed at preoperative and postoperative follow-ups according to neck symptom improvement status and neurological functional recovery status.^8^ Preoperative and postoperative axial symptom improvement status was evaluated according to the neck disability index (NDI), which uses scores ranging from 0 (no dysfunction) to 50 points (complete dysfunction). Recovery of neurological function was scored according to the Japanese Orthopedics Association Scoring System (JOA)^9^ 17-point method.

### Statistical Methods

SPSS18.0 statistical software package was used for analysis. Data were presented as means ± standard deviation (SD). Preoperative and postoperative CI, ROM changes, JOA score, and NDI score were analyzed by using paired *t*-tests. A paired *t*-test was conducted to statistically analyze the difference between the preoperative and postoperative score changes. A *P* value of <0.05 was considered statistically significant.

## RESULTS

The operations on 60 patients in this group were completed by the same group of surgeons. The mean operation time was approximately 100 minutes, and the mean blood loss was approximately 200 ml. All surgical patients received follow-up for 18 to 24 months, with an average of 20 months and all achieved satisfactory clinical efficacy.

### Imaging Evaluation

**FIGURE 5:**
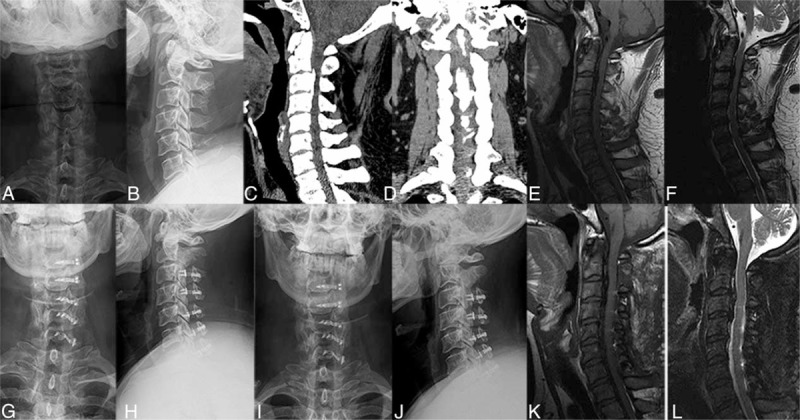
OPLL: A 37-year-old male patient (A-L), preoperative X-ray (A, B), CT (C, D), MRI (E, F), 1 week (G, H), and 1 year (I, J) after operation the X-ray showed good internal fixation; 1 year after operation the MRI examination (K, L) showed spinal cord without compression. OPLL =  ossification of posterior longitudinal ligament.

**FIGURE 6:**
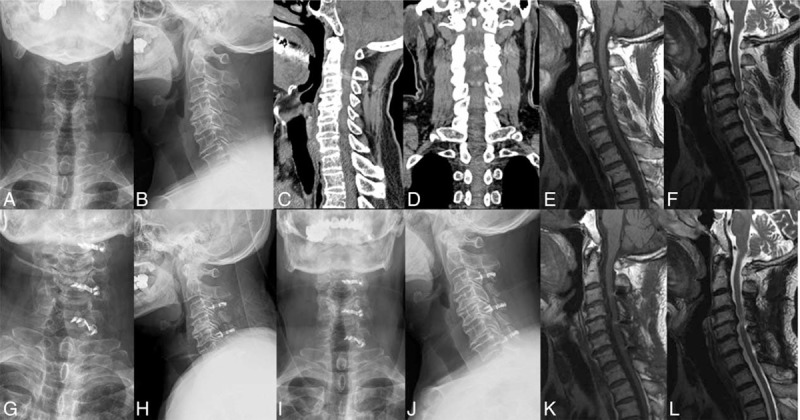
CSM: A 63-year-old male patient (A-L), preoperative X-ray (A, B), CT (C, D), MRI (E, F), 1 week (G, H), and 1 year (I, J) after operation the X-ray showed good internal fixation; 1 year after operation the MRI examination (K, L) showed spinal cord without compression. CSM = cervical spondylotic myelopathy.

All patients’ imaging parameters were measured by the same researcher from my department. No screw looseness, shifting, or titanium miniplate fractures were discovered in any patients during follow-up. Cervical lordosis did not significantly decrease compared with that before operation, and no kyphosis deformity occurred. Preoperative CI was 10.49% ± 3.93%, and compared with postoperative 14.14% ± 2.85% the difference was statistically significant (t = 8.07, *P* < 0.05). Postoperative ROM in cervical spine decreased compared with that before operation. Specifically, it decreased from preoperative 43.35° ± 7.55° to postoperative 34.83° ± 7.41°. The difference was statistically significant (t = −6.78, *P* < 0.05), but no patient had a completely stiff cervical spine (Table 1). A postoperative MRI scan showed that spinal cord compression disappeared and the spinal canal significantly expanded. Typical cases are shown in Figures 5 and 6.

**TABLE 1:**
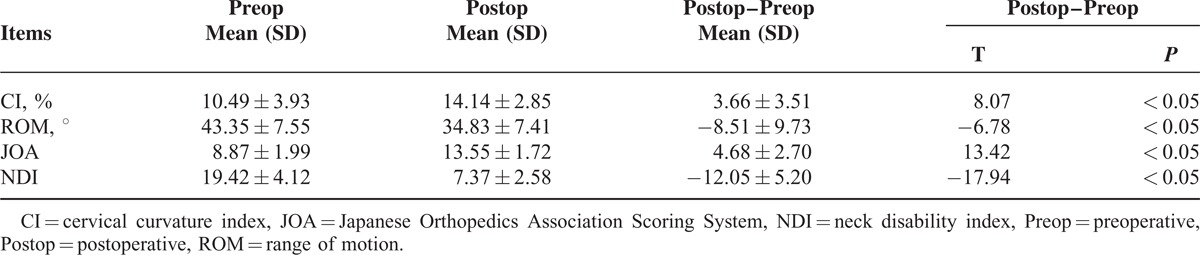
Comparisons of Preoperative and Postoperative Imaging and Clinical Function Evaluations (x¯ ± s, n = 60

### Evaluation of Clinical Function

The spinal nerves functions of all patients were significantly improved, of which among 12 patients with preoperative axial symptoms, 8 patients were relieved and the remaining 4 patients had no significant change. All patients without preoperative axial symptoms had no postoperative axial symptoms. JOA score increased from preoperative 8.87 ± 1.99 to 13.55 ± 1.72 at last follow-up, and the difference was statistically significant (t = 13.42, *P* < 0.05). Postoperative NDI was 7.37 ± 2.58 and significantly decreased compared with preoperative 19.42 ± 4.12 (t = −17.94, *P* < 0.05) (Table 1).

### Surgical Complications

Any postoperative complication was recorded, only 1 patient had postoperative C5 nerve root palsy and completely recovered 1 month later. No neurological functional aggravation, no infection, or other complication occurred.

## DISCUSSION

Expansive open-door cervical laminoplasty was first proposed by Hirabayashi et al^10^ in 1976. As surgical techniques are continuously being improved and surgical devices become increasingly fine, the rate of good surgical efficacies continues to improve while surgical time, blood loss, and relevant complications decrease.^11,12^ However, after operations a variety of symptoms remain or occur, including neck and shoulder pain, stiffness, and limited neck motion.^13^ We believe that expansive open-door cervical laminoplasty creates a relative large injury and will cause damage of the ligament complex to various degrees when relaxing pressure. Because attachment relationships between posterior cervical muscular attachment points and spinous process cannot be reconstructed, posterior cervical extensors become atrophic and weak; in addition, postoperative long-time neck immobilization will cause neck symptoms. We took a conventional surgical approach and used in situ reconstruction of extensor muscle insertion on the C2 spinous process to minimally decrease damage and to balance muscles on both sides of the neck. Operation duration was less than 100 minutes and blood loss did not exceed 200 ml. Therefore, surgical injury was minimally decreased and the patients’ postoperative rehabilitation was promoted. C3–C7 spinous processes were removed by a pair of rongeurs to avoid postlaminoplasty local distance widening and heightening, ensuring semispinalis cervicis (SSC) bundle traveling would not be affected. Early postoperative rehabilitation exercise allowed patients to obtain satisfactory clinical efficacy.

Normal cervical curvature and sagittal plane mechanical balance are the basis to maintain normal ROM in the cervical spine. Furthermore, sagittal plane mechanical balance and cervical curvature are maintained by the best tension of posterior cervical muscles and ligaments. In an electrophysiological experiment, Moroney et al^14^ found that when normal persons completed extreme cervical flexion, extension, and other motions, neck electromyogram had no significant change. This indicated that no cervical muscle load significantly changed and that the ligament tissue bore most of the increased load. In expansive open-door cervical laminoplasty, ligament continuity is inevitably damaged; if extensor groups are also seriously injured, cervical spinal sagittal sequence often cannot be effectively maintained, lordosis decreases, and degeneration intensifies. The study of Fujibayashi et al^15^ also has shown that decrease of intraoperative damage to cervical muscles can decrease incidence of postoperative axial symptoms, and that the cervical muscle strength is negatively related to the degree of postoperative neck pain. Therefore, intraoperative protection of posterior cervical muscles is particularly important.

Vasavada et al^16^ measured neck extension power devices in a 3-dimensional biomechanical model, and found that 37% of extension arm was generated by SSC. Therefore, if SSC repair is poor, biomechanical dysfunction will be caused, often leading to muscle fatigue and pain, which further aggravates the imbalance of cervical spinal mechanics. The experimental study of Takeshita et al^17^ thought that in expansive open-door laminoplasty, after retaining muscle tissue attached to C2 spinous process, C2–C7 sagittal plane intervertebra angle change was only an average of 1.5°, which played a significant role in stabilizing the cervical spine. Therefore, in situ reconstruction of extensor muscle insertion on the C2 spinous process is vital. However, suture reconstruction is difficult after muscular insertion of C2 is freed using traditional surgical methods. Even if suture reconstruction of SSC and other neck extensor groups is performed, there are still 18% of patients with poor postoperative extensor functional repair, even leading to occurrence of postoperative cervical kyphosis deformity.^18^ We found that if muscular insertion was intentionally retained, the difficulty of operation would be significantly increased and operation duration would be significantly prolonged.

Anatomical studies have found interesting data. The SSC attachment site on C2 spinous process has an average width of 10.6 mm, and because it is significantly different due to different spinous process sizes and opening angles, suture reconstruction is difficult to achieve at the exact anatomical location, which affects mechanical function and may be one reason for postoperative poor repair.^19^ We routinely use a 1 mm burr to cut off the partial bone of C2 spinous process attaching muscles and make the retained bilateral cortices symmetric. Furthermore, we retain the partial bone attaching muscles as much as possible to facilitate postoperative in situ reconstruction. We have found that if the retained cortex is too little, avulsion of muscle attachment points easily occurs during reconstruction. In addition, we use a spinous process punch to drill before osteotomy so that accuracy of postoperative in situ reconstruction is better ensured to maximize recovery of neck extensor function.

Titanium miniplates internal fixation can be used to instantly reconstruct the spinal canal to restore its integrity. A relatively stable mechanical environment is conducive to bone slot healing on the door axial side and is also conducive to postoperative left and right muscle strength balance in the neck. This balance means that during 3-dimensional motion of the cervical spine the load distribution on both sides is coordinated to avoid excessive fatigue of muscles on one side. Studies have also found that when a simple titanium miniplate is used to fix laminas and lateral masses on the open-door side, stability can be provided at once and a patient can perform early postoperative activity. In addition, compared with bone union, the thin profile of a titanium miniplate provides more space for the spinal canal, assisting decompression. Finally, it is found that a titanium plate has good toughness and will not break.^20^

We believe that titanium miniplates internal fixation can avoid the muscle spasms and pain caused by suturing facet joint capsules, and can avoid the inflammation caused by subsequent facet joint injury. In addition, scar contracture of the facet joint capsule is an important cause for decrease of ROM in cervical spine and is obviously related to the postoperative decreased ROM and the aggravated axial symptoms. Therefore, although titanium miniplates fixation restores the integrity of the spinal canal, it also avoids facet joint capsule injury, decreases postoperative scar adhesions, and theoretically decreases incidence of axial symptoms.

In summary, this group of patients received a conventional surgical approach, and by using in situ reconstruction of extensor muscle insertion on the C2 spinous process, the most important extension power muscles of the cervical spine were retained to provide the possibility of early postoperative functional exercise. Additionally, the process avoided possible rupture of SSC and other power muscles during functional exercise. Through titanium miniplates inner fixation, a relatively complete spinal canal and a relatively stable door on the axial side were established, so that postoperative long-term immobilization was unnecessary, decreasing muscle atrophy and joint/ligament contracture possibilities. In this group of cases, spinal nerves functions of all patients were significantly improved. All patients without preoperative axial symptoms had no postoperative axial symptoms. It is obviously declined than Ratliff and Cooper's^21^ report that the axial symptoms occur rate is 6% to 60%. The ROM in cervical spine has a decrease from preoperative 43.35° ± 7.55° to postoperative 34.83° ± 7.41°. The differences were of statistical significance, but compared with the Heller et al's^22^ report that the ROM loss is 30% to 70%, it has been obviously improved.

We believe expansive open-door cervical laminoplasty, in situ reconstruction of extensor muscle insertion on the C2 spinous process combined with titanium miniplates internal fixation is a safe and effective treatment method, and can effectively reduce postoperative complications and achieve satisfactory clinical efficacies. Strictly mastering indications and mastering the timing of the operation is vital to the process.
